# The efficacy of a compounded micronutrient supplement on the incidence, duration, and severity of the common cold: A pilot randomized, double-blinded, placebo-controlled trial

**DOI:** 10.1371/journal.pone.0237491

**Published:** 2020-08-25

**Authors:** James G. Lenhart, Phuong T. Vu, Kale Quackenbush, Anne LaPorte, Jeff Smith

**Affiliations:** 1 Community Health Care Family Medicine Residency, Tacoma, Washington in affiliation with the Family Medicine Residency Network, University of Washington School of Medicine, Seattle, Washington, United States of America; 2 Department of Biostatistics, School of Public Health, University of Washington, Seattle, Washington, United States of America; University of Ghana, GHANA

## Abstract

**Purpose:**

Viral upper respiratory infections are associated with significant health and economic impact. This study sought to determine the efficacy of routine immune system micronutrient supplementation on the incidence, duration and severity of common cold symptoms.

**Methods:**

This pilot study was a randomized, double-blinded, placebo-controlled trial of N = 259 with asymptomatic participants aged 18 to 65 in two cold seasons of 2016 and 2017. The treatment group received an immune system targeted micronutrient caplet, while the placebo group received a micronized cellulose caplet externally identical to the treatment caplet. Weekly surveys were sent electronically to participants to document common cold incidence, duration and severity. Primary statistical results were obtained using mixed-effects logistic regressions to account for longitudinal measurements for participants.

**Results:**

The odds of acquiring an upper respiratory infection, adjusted for potential confounders, was estimated to be 0.74 times lower in the treatment group (p = 0.14). The odds of reporting specific symptoms were statistically lower in the treatment arm compared to the placebo arm for runny nose (OR = 0.53, p = 0.01) and cough (OR = 0.51, p = 0.04). Shorter durations of runny nose and cough were also observed in the treatment arm compared to placebo (both p < 0.05). There was no significant difference in severity of symptoms in either group. The observed proportion of reported cold symptoms in the treatment group was lower compared to the placebo group between late January and February in two consecutive cold seasons. Given the physical, workplace and economic impact of upper respiratory infections, this low cost and low risk intervention should be further studied with more robust investigation and meticulous experimental design.

## Introduction

The common cold has been previously defined as a viral upper respiratory infection (URI) with a compilation of symptoms including runny nose, postnasal drip, cough, congestion, headache, body aches, and pharyngitis (sore throat), but rarely high fever. It is thought to be the most common acute illness throughout the industrialized world [1].

The average US adult has 2 to 4 colds per year lasting 5 to 7 days [2]. According to Bramley’s analysis, colds experienced by working adults annually cause an average of 8.7 lost work hours/cold, which includes 2.8 hours in absenteeism and 5.9 hours of on the job losses [3]. The financial impact is significant: $16.6 billion lost in job productivity, $8 billion lost in absenteeism plus caregiver absenteeism totaling $230 million which equates to an estimated annual economic loss of nearly $25 billion [3]. Fendrick’s findings (2003) showed the economic impact at the time of $17 billion in direct costs (physician visits, medications, and other treatments) and $22.5 billion in indirect costs (lost time from work and job productivity) [4]. More recently, the ACHOO survey in 2015 revealed the majority of male and female respondents had at least one or more colds per year with 52% of participants declaring it impacted work life leading to absenteeism (44.5%) and decreased productivity (26.4%). As well, 93% of respondents reported significant sleep difficulty due to cold symptoms, which impacted preabsenteeism and absenteeism [5]. Hellgren, et al (2010) analyzed the societal cost of allergic rhinitis and the common cold estimating 5.1 days of absenteeism at a cost of euro 653 per worker per year (total productivity loss in Sweden of euro 2.7 billion) [6].

Protein-energy and micronutrients play crucial roles in the development and maturation of innate and adaptive immunity; micronutrient deficiencies in vitamins A, D and E as well as minerals zinc, selenium and iron can lead to immunosuppression and susceptibility to infection [7–9]. Malnutrition is considered to be the primary cause of immunodeficiency worldwide with micronutrient deficiencies effecting growth, intellectual development, infant and childhood mortality and susceptibility to infection [8].

Food insecurity persists throughout the world in both industrialized and developing countries. In 2011, it was reported that 20.1% of US families with children met criteria for food insecurity [5]. According to data from the National Health and Nutrition Examination Survey 2013, significant percentages of the US population do not meet the estimated average daily nutritional requirements for the following micronutrients: 93% for Vitamin E, 44% for Vitamin A, 31% for Vitamin C, 14% for Vitamin B6, and 12% for Zinc [10], which places them at risk for immune system deficiencies. Furthermore, Vitamin D deficiency is a nutritional predicament in the US and internationally; it has been estimated that one billion people worldwide have either vitamin D deficiency or insufficiency [11].

Evidence on the potential relationship between micronutrient supplementation and the incidence, severity and duration of the common cold is inconsistent. For example, studies conducted on elderly and nursing home populations concluded no benefit [12–14]. Whereas research focusing on working aged adults suggested that supplementation may be beneficial, especially in diabetics [15–17]. In the recent decade, studies have been conducted on the efficacy of various micronutrients to reduce the frequency, duration, and severity of the common cold symptoms, yet the results remain inconclusive [18–31]. These studies have focused on the effect of individual micronutrients, or in some limited combinations. Factors which have restricted conclusive outcomes include study design, sample size, recruitment and retention [32]. In particular, there is a lack of reliable observational studies or well-designed clinical trials on multi-ingredient supplements [32].

This study sought to determine the efficacy of micronutrient supplementation, with a formulation targeting the immune system function, on the incidence, duration and severity of common cold symptoms (sore throat, headache, runny nose, cough, congestion and aches). The investigators hypothesized that regular consumption of an immune system targeted micronutrient supplement would decrease the incidence, duration and severity of common cold symptoms.

## Methods

### Study design

Researchers conducted a pilot study that was designed as a randomized, double-blinded, placebo-controlled trial of an immune system micronutrient supplement against placebo relative to the incidence, duration and severity of the common cold amongst working men and women aged 18 to 65 years.

Study subjects were drawn from non-smoking, working males and females aged 18 to 65 years from the five federally qualified health centers in the south Puget Sound region affiliated with the Community Health Care Family Medicine Residency Program. Exclusion criteria (included in S1 Appendix) included currently smoking, pregnancy, breast feeding, a history of adverse effects to vitamin and mineral supplements, gastrointestinal surgery, immune deficiency or suppression, inflammatory bowel disease, malabsorption syndromes, gastrointestinal dysfunction and current use of disease modify in anti-rheumatic drugs. Informed consent was obtained prior to enrollment and study participants were incentivized to improve survey completion.

All principal investigators provided current evidence of human subjects’ research certification as a requirement of their engagement. The study was scrutinized and approved by the Catholic Health Initiatives (CHI) Franciscan Medical Research Evaluation Committee (MREC), which functions as the institutional review board (IRB) for the Franciscan Health System in the greater Puget Sound region. The MREC provided approval on October 1, 2015 and re-approval on August 23, 2016 (reference number CHC012016).

The study was registered with ISRCTN on December 28, 2018, after the study was completed (registration number ISRCTN68878070). The investigators acknowledged our misinterpretation of the Food and Drug Administration Amendments Act of 2007 which requires registration and report of results for certain clinical trials of drugs, biologics, and devices that are subject to FDA regulation (Public Law 110–85, Title VIII). The investigators pursued this study with the understanding that FDA registration was not required, as micronutrient supplements are not regulated by the FDA. This unfortunate oversight was uncovered during initial submission for peer reviewed publication. Furthermore, the authors confirm that all ongoing and related trials for this drug/interventions are registered.

Randomization assignments were generated using permuted block randomization, using random block sizes of 4, 6, or 8. Study participants were randomized into two groups, where both the participant and research staff were blinded to the assignment. One group consumed the active immune system targeted multivitamin and mineral micronutrient supplement, while the other group consumed a micronized cellulose placebo with physical characteristics identical to the active caplet. The randomization and study procedure are described in S1 Appendix. The compositions of the active and placebo caplets are described in S2 Appendix. A survey instrument designed to identify and capture critical data was developed and integrated into the Research Electronic Data Capture (REDCap) [33] to enhance data capture and analysis. Important questions used in the survey are included in S3 Appendix. Surveys were sent out electronically on a weekly basis.

The study was designed to be a pilot study in order to better inform a larger trial. While IRB approval was obtained for a much larger number of participants, the sample size was pre-specified to be 250 participants in total due to financial and logistic constraints. Data were collected during two common cold seasons from the beginning of January through the end of March in consecutive years 2016 and 2017 utilizing identical study protocols, as the pre-specified sample size was not reached in the first cold season. Participants were screened and enrolled in December of 2016 and 2017. Weekly surveys were sent to participants from January 4, 2016 through March 28, 2016, and from January 2, 2017 through March 27, 2017. Data from 61 participants surveyed in 2016 and 198 participants surveyed in 2017 were combined and included in the statistical analysis. Throughout each trial, study subjects were instructed not to take any additional multivitamin and mineral supplements.

### Micronutrient supplementation

The formula of the micronutrient supplementation utilized in this study was designed to target the immune system, which included vitamins A, D, C, E, B6, B12, folic acid, zinc, selenium, copper, and iron.

Both innate and adaptive immunity depend upon vitamin A. It is essential in the maintenance of mucosal barrier functions and the function of natural killer cells, macro phages, neutrophils, humoral immunity and B lymphocytes. Vitamin A supplementation has been shown to enhance immunity [34–36]. Similarly, vitamin D has been shown to improve immune cell proliferation and cytokine production. The beneficial effects of vitamin D supplementation have been widely studied with varying results [36–38].

Vitamin C acts as an effective antioxidant to combat free radical production. Vitamin C stimulates white blood cell formation including lymphocytes and neutrophils. It has been shown to play a role in antibody development and complement proteins [39]. Like vitamin C, vitamin E has effective antioxidant properties. In this capacity, vitamin E plays a role in T lymphocyte (killer cell) and B lymphocyte (antibody) proliferation and function [40, 41].

Vitamin B6 deficiency effects adaptive immune system activity lowering cytokine and T lymphocyte proliferation and function, however, few studies have been performed to demonstrate supplement benefit [42–44]. Vitamin B12 modulates immune function through its effect on T lymphocytes and natural killer cell activity, and therefore plays a crucial role in adaptive immunity [45, 46].

Folic acid plays a role in the metabolism of DNA and RNA. At this very basic genetic level, it is integral to immune system function. It is thought to primarily affect cell mediated immunity, and has been shown to improve immune system function [47]. Selenium is required for selenoprotein dependent enzymes, which are necessary for antibody production and antioxidant activities. In addition, it plays a role in natural killer cell lymphocyte function and interleukin 2 receptor expression [48–51]. Copper and its relationship to the immune system is not clearly understood. Although thought to be a critical micronutrient for immune system function, exact mechanisms are unclear. It is thought that interleukin 2 and copper interact to affect improved T cell proliferation [52].

Zinc’s role in immune health has been widely studied and supplementation has been shown to improve immune system performance, while deficiency leads to reduced immune system performance in particular affecting T lymphocyte proliferation and function [53–56]. Although iron plays a role in oxygen transport and oxidative metabolism and immunity, iron has been shown to interfere with the absorption of zinc [57, 58]. Due to zinc’s established benefit on the duration of common cold symptoms, iron was not included in the designed formula utilized in this investigation.

The investigators arrived at the micronutrient ingredient quantity in each caplet based on an initial value consistent with 100% of the Recommended Daily Allowance (RDA) adjusting it up or down based upon literature review of micronutrient-URI studies, Tolerable Upper Intake Levels (TUL), and fat solubility [59].

### Statistical analysis

The primary outcome of interest was the odds of developing an upper respiratory infection (URI). We compared the odds of acquiring these symptoms between the treatment and placebo groups using a mixed-effects logistic regression model to account for longitudinal measurements over 12 follow-up time points for each participant [60]. We also adjusted for a linear trend over time and difference in calendar years.

Our secondary outcomes of interest were the incidence, duration, and severity of specific symptoms: sore throat, headache, runny nose, cough, congestion, aches, and fever. Since fever with body temperature higher than 100.4°F is not a classic symptom or sign of the common cold, data associated with episodes of fever (T>100.4°F) (four observations) were excluded from all analyses of common cold symptoms.

To assess the incidence per symptom, we modeled the odds of symptom between the treatment and placebo arms. Those who did not report a URI were assumed to have none of the seven symptoms. We used the same mixed-effects logistic regression framework that adjusted for time trend and difference in calendar years.

Those who reported any of the seven symptoms were then instructed to answer questions about duration. The survey provided three options for duration: less than 24 hours, between 24 and 48 hours, and greater than 48 hours. We converted the categories into a numerical system with conservative assumptions. We assumed that “less than 24 hours”, “between 24 and 48 hours”, and “greater than 48 hours” were equivalent to 12 hours, 36 hours, and 48 hours respectively. This was a conservative assumption, as the actual duration for those with “greater than 48 hours”, for example, could vary anywhere between 48 hours and 7 days. Those who did not report a URI or did not report a symptom were assumed to have duration of 0 hours for that specific symptom. We compared the mean weekly duration between the treatment and placebo groups using a mixed-effect regression model to account for longitudinal measurements over 12 follow-up time points for each participant, with adjustments for linear trend over time and difference in calendar years. This is consistent with the statistical model used for our primary analysis.

We utilized the same approach for analysis of symptom severity. Except for fever, there were three options for severity: mild, moderate, and missed work or play. To be conservative, we assigned these categories with scores of 0, 1, 2, and 3, respectively. Those who did not report a URI or did not report a symptom were assumed to have a severity score of 0 for that specific symptom. Data related to episodes with fever greater than 100.4°F were excluded from analysis as temperature elevations greater than 100.4°F are not consistent with the diagnosis of URI. As a result, those who reported fever of less than 100.4°F were given a score of 1. Similar to the analysis on duration, we compared the mean weekly severity score between the treatment and placebo groups using a mixed-effect regression model to account for longitudinal measurements over 12 follow-up time points for each participant, with adjustments for linear trend over time and difference in calendar years.

Due to the imbalance between the numbers of male and female participants unresolved via randomization, we later conducted a sensitive analysis in which all of the above models were re-run with an additional adjustment for gender. The sample size was slightly reduced for these analyses, as there were four participants who had missing data on gender.

Statistical significance was defined as 0.05. All statistical analyses were performed using the R statistical analysis package version 3.4.1 [61]. Research coordinators maintained all data in the REDCap database [34]. The Center for Biomedical Statistics at the University of Washington hosted an independent REDCap database to securely store participant survey responses as well as randomized treatment assignments.

## Results

**Fig 1:**
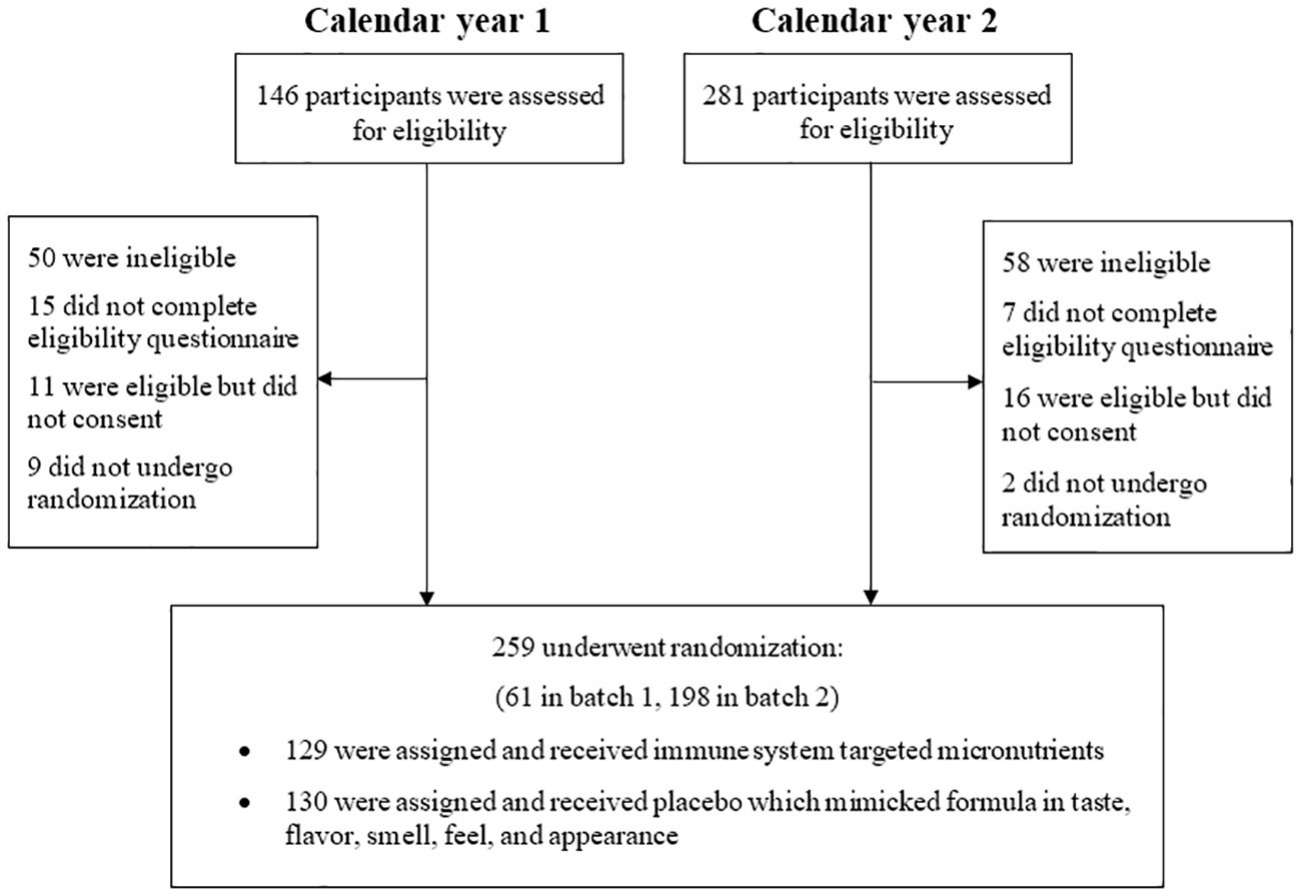
Flow chart of selection, randomization, and follow-up of participants approached and included in the analysis.

Fig 1 provides a flow diagram of the number of participants initially approached, excluded, selected, randomized, and eventually included in the statistical analysis. All N = 259 randomized participants were surveyed weekly for 12 weeks. The response rate was 80.3% in the first week, and dropped down to 59.8% in the final week, which led to an average of 66.2% across the 12 weeks. The average self-reported number of caplets taken per week for a participant throughout the follow-up period was 6.03, with a standard deviation (SD) of 1.60. The follow-up rates were similar over time between the two treatment groups, as shown in the S4 Appendix.

Data were collected over two cold seasons from the beginning of January through the end of March. The first set of data was collected in 2016 and the second set of data was collected in 2017. Data from 61 participants surveyed in 2016 and 198 participants surveyed in 2017 were included in the statistical analysis. The baseline characteristics of these 259 participants are shown in Table 1. While we expected the randomization procedure to alleviate any confounding issues, the gender distributions were not similar between the placebo and treatment arms (p = 0.04). This motivated a sensitivity analysis in which all models were re-run with an additional adjustment for gender.

**Table 1:** Baseline characteristics of study participants.

Baseline characteristics	All participants ^[1]^ (N = 259)	On placebo (N = 130)	On treatment (N = 129)	P value^[2]^
Age (years)—mean (SD)	39.3 (12.7)	39.0 (11.7)	39.5 (13.7)	0.76
Male—# (%)	89 (34.4%)	52 (40.0%)	37 (28.7%)	0.04
Live with children less than 12 years old—# (%)	97 (37.5%)	53 (40.8%)	44 (34.1%)	0.37
Type I diabetes—# (%)	2 (0.8%)	1 (0.8%)	1 (0.8%)	0.32
Type II diabetes—# (%)	10 (3.9%)	4 (3.1%)	6 (4.7%)	0.08
Diet type				0.62
No restriction—# (%)	213 (82.2%)	108 (83.1%)	105 (81.4%)	
Vegetarian—# (%)	12 (4.6%)	6 (4.6%)	6 (4.7%)	
Fish, no other meat—# (%)	3 (1.2%)	1 (0.8%)	2 (1.6%)	
Other—# (%)	17 (6.6%)	6 (4.6%)	11 (8.5%)	
Take regular multivitamin or micronutrient supplement—# (%)	76 (29.3%)	31 (23.8%)	45 (34.9%)	0.07
Have flu vaccine this year—# (%)	121 (46.7%)	63 (48.5%)	58 (45.0%)	0.39

^[1]^ The summary statistics were based on complete baseline data. There were missing data on baseline age (6), sex (4), type I diabetes (5), type II diabetes (8), diet type (14), whether participant took regular multivitamin or micronutrient supplement (6), and whether participant had a flu shot within that cold season (5).

^[2]^ P-values presented were from testing differences in baseline characteristics between treatment and placebo arms.

**Fig 2:**
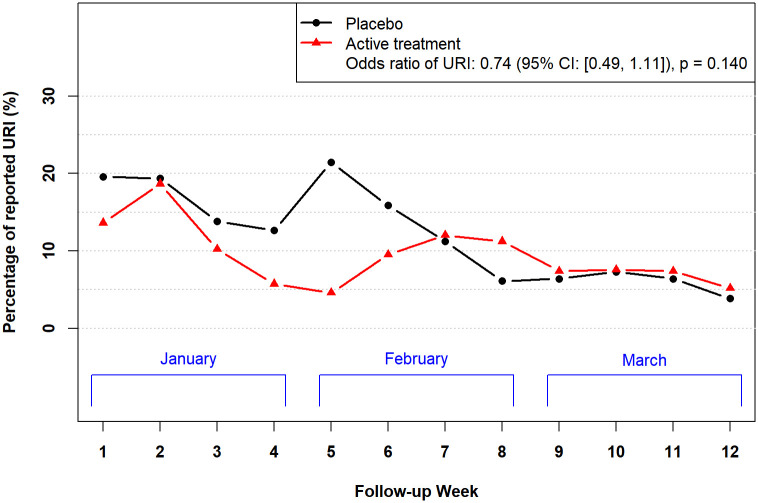
The proportions of participants reporting a URI among those who completed the weekly surveys. The ORs of reporting URI between the treatment arm and the placebo arm was obtained from a generalized linear mixed model adjusting for possible temporal trend, follow-up calendar year, and multiple responses per participant.

Fig 2 shows the proportions of participants reporting URI out of those who responded to the weekly surveys. The odds of acquiring a URI (adjusting for time and potential difference in calendar years) was estimated to be 0.74 times lower in the treatment group, with a 95% confidence interval (CI) for the odds ratio (OR) being [0.49, 1.11] (p = 0.14).

While this primary result of interest was not statistically significant, there was a noticeably lower proportion of reported URIs in the treatment group between late January and early February. As an exploratory analysis, we fitted the same model on only data collected in the last two weeks of January and the first two weeks of February. The odds of acquiring a URI, adjusting for time and potential difference in calendar years, was estimated to be 0.40 times lower in the treatment group, with a CI of [0.20, 0.78] (p = 0.01). While this was a subset analysis outside of the primary result of interest, this phenomenon was observed in both sets of data, as illustrated in Fig 2 and the S5 Appendix.

Table 2 shows the ORs of reporting a specific symptom between the treatment and placebo groups for all seven symptoms of interest. The odds of reporting the frequency of specific symptoms was statistically significant for runny nose and cough.

**Table 2:** Odds ratios of reporting a symptom between the treatment and placebo groups for seven symptoms of interest.

Symptom	Odds ratio ^[1]^	95% Confidence interval	P-value
Sore throat	0.79	[0.47, 1.32]	0.37
Headache	0.62	[0.31, 1.22]	0.17
Runny nose	0.53	[0.33, 0.85]	0.01
Cough	0.51	[0.27, 0.96]	0.04
Congestion	0.63	[0.39, 1.03]	0.07
Aches	0.98	[0.44, 2.02]	0.95
Fever	0.62	[0.29, 1.34]	0.23

^[1]^ The OR of reporting a symptom between the treatment arm and the placebo arm was obtained from a generalized linear mixed-effect model adjusting for possible temporal trend, follow-up calendar year, and multiple responses per participant.

**Fig 3:**
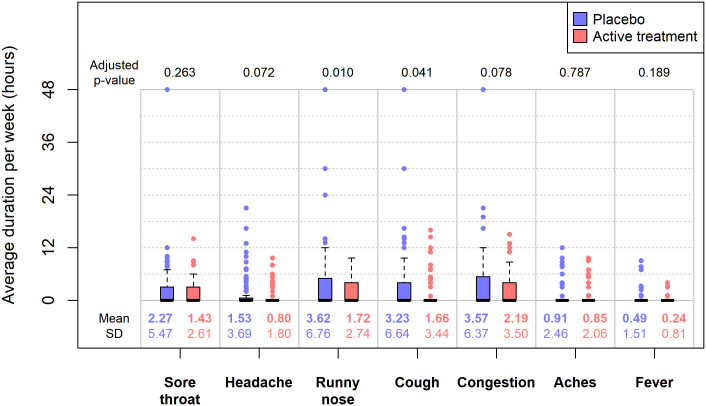
The weekly durations between two treatment groups by symptom. Data for the placebo arm are in black while data for the treatment arm are in red. For each of the seven symptoms of interest, the p-values were obtained by comparing the mean differences of weekly durations between two treatment groups, using mixed-effect regression models with adjustments for multiple responses per participant, linear trend in time, and difference in calendar years.

**Fig 4:**
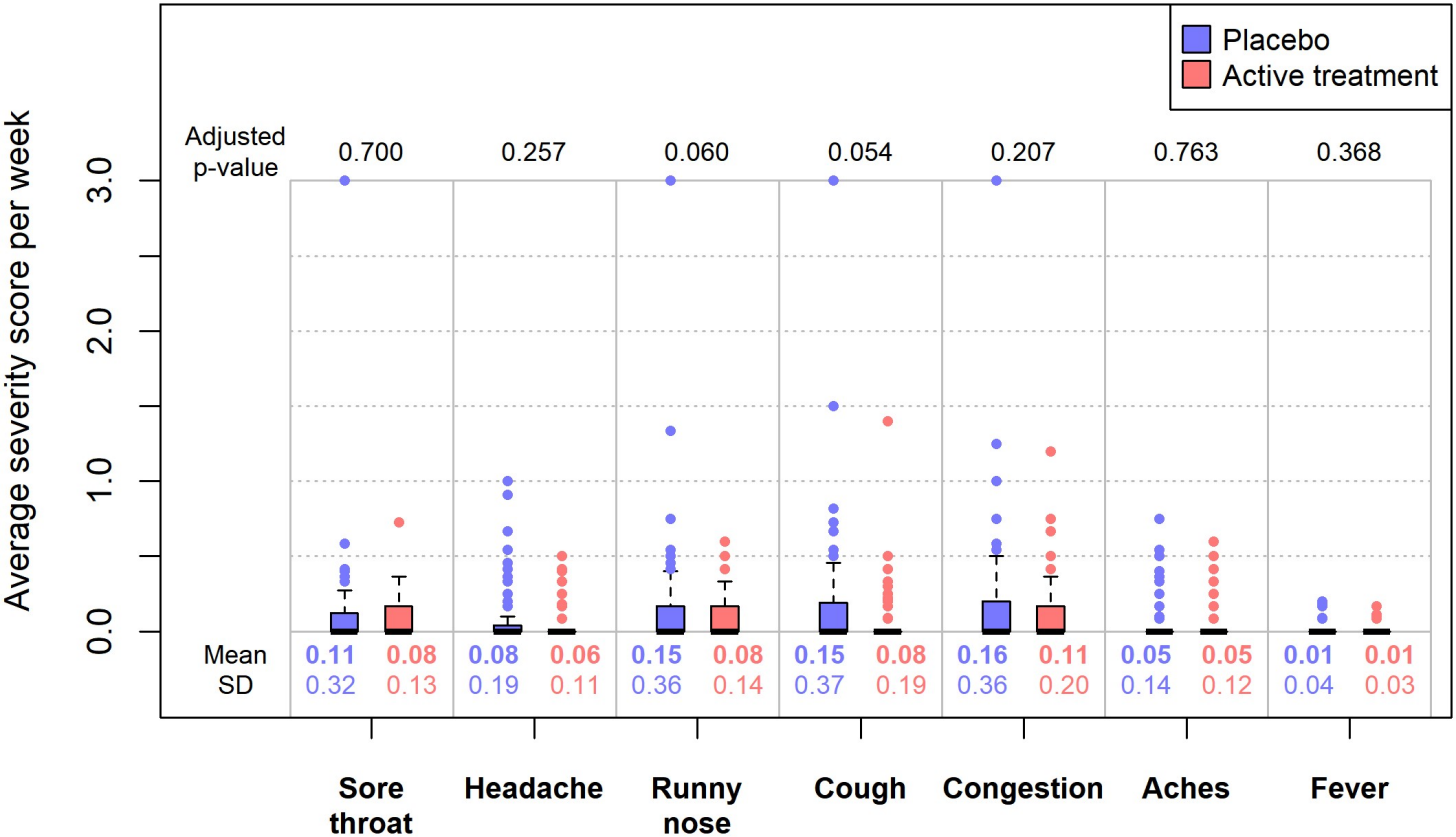
The weekly severity scores between two treatment groups by symptom. Data for the placebo arm are in black while data for the treatment arm are in red. For each of the seven symptoms of interest, the p-values were obtained from comparing the mean differences of weekly severity scores between two treatment groups, using mixed-effect regression models with adjustments for multiple responses per participant, linear trend in time, and difference in calendar years. Note that individual severity score for fever had only two levels (0 or 1), while severity score for the other six symptoms had four levels (0, 1, 2, and 3).

Figs 3 and 4 show the statistical results for the average weekly durations and severity scores across the two groups by each symptom of interest. In these figures, we also provided the unadjusted means and standard deviations of the weekly durations or severity scores by treatment group, i.e. the average duration (in hours) or severity score *per person per week* across the 12-week follow-up period. Utilizing our conservative scoring approach, the mean weekly durations were estimated to be significantly lower in the treatment group compared to the placebo group for runny nose (3.62 hours versus 1.72 hours, p = 0.01) and cough (3.23 hours versus 1.66 hours, p = 0.04). The duration results were not statistically significant for the remaining symptoms. We did not observe any statistically significant difference in mean weekly severity scores between the two treatment groups for any symptom.

In the sensitivity analysis, we refitted all above models with an additional adjustment for gender. This analysis only included data from 255 individuals with no missing data on gender. Adjusting for temporal trend, potential difference in calendar years, and gender imbalance, the odds of acquiring a URI, our primary outcome of interest, was estimated to be 0.71 lower in the treatment group (95% CI of [0.47, 1.08], p = 0.114). Results on incidence, duration, and severity by symptoms are reported in S6 Appendix. Mean differences between treatment groups for duration of cough, as well as severity scores of runny nose and cough, became statistically significant when adjusting for differences in gender distributions.

## Conclusions and discussions

This investigation sought to determine the efficacy of regularly consumed micronutrient supplementation, with a formula targeting the immune system, on the incidence, duration and severity of common cold symptoms. Published studies in the recent decade offer conflicting results and inconsistent evidence for benefit [18–31] and often focus on individual vitamins or combination of a small number of vitamins [32]. Designed as a double-blinded, randomized clinical investigation on the efficacy of a supplement formulated with multiple vitamins and minerals, our pilot study 1) utilized a multivitamin and mineral supplement engineered with micronutrients essential to immune system function, 2) studied the efficacy of the micronutrient formulation over two consecutive peak URI seasons, and 3) investigated the effect on working adults aged 18 to 65 years.

The odds of acquiring a URI (adjusting for time and potential difference in calendar years) were estimated to be 0.74 times lower in the treatment group. While this primary result of interest was not statistically significant, there was a noticeably lower proportion of reported URIs in the treatment group between late January and early February. Subset analysis based on the data collected in the last two weeks of January and the first two weeks of February showed significant lower odds of acquiring a URI in the treatment group compared to the placebo group.

The odds of reporting specific symptoms were significantly lower in the treatment arm compared to the placebo arm for two symptoms, runny nose and cough. Shorter durations of runny nose and cough were observed in the treatment arm compared to placebo (p <0.05), as well. There was no significant difference in severity of symptoms in either group. The primary results did not change when the statistical models accounted for gender in our sensitivity analysis.

Some potential limitations in the study design and data collection included the relatively small sample sizes and the fact that follow-up was done in two different calendar years, which was unavoidable given available resources. Although the investigators hoped to enroll as many as 1000 participants, a pre-specified sample size of 250 was targeted instead, due to financial and logistic constraints. We attempted to mitigate the impact of these limitations by combining data from follow-up periods to gain more power in detecting signals, while adjusting for potential difference in calendar years appropriately in our statistical models. While combining two cohorts followed over two cold seasons was not ideal, this can be justified by the identical protocols and similar study populations drawn from the same geographic region.

Another limitation in the study design was the lack of separating the common cold from any form of the flu. There is a potential bias given the similarity in symptoms of both ailments and the inclusion of participants that had received flu vaccine. While not ideal, we attempted to correct for such bias by excluding data associated with fever of body temperature higher than 100.4F. Randomization also assured that the proportion of participants who got flu vaccine was similar between the two treatment groups, and therefore would not confound the overall results. For future studies, more stringent eligibility criteria, including flu vaccination or testing for flu, should be in place for more robust results that are specific to the common cold.

Randomization unfortunately failed to achieve similar gender distributions in the two treatment arms. We corrected for gender balance in our sensitivity analyses. Significant results from our original analyses still remained statistically significant even with the added adjustment of gender in all models. Intriguingly, we obtained more statistically significant results for duration and severity analyses when adjusting for gender. This suggests that future studies could potentially be improved by considering gender impact.

Modeling limitation also pertained to our secondary analysis, as data on duration and severity were collected as categorical data. With such data, mixed-effect, ordered, multinomial regressions would be the most appropriate, however, it was not feasible due to the small number of reported symptoms and the number of variables to be adjusted for. While converting ordered categories in a numerical system would not be statistically ideal, the conversion was carefully done with conservative assumptions.

In this study, there is some evidence that a multivitamin and mineral supplement engineered with micronutrients essential to immune function reduces the frequency and duration of URI symptoms. The incidence and duration of runny nose and cough were significantly lower in the treatment group. Noticeably, lower proportions of reported URIs in the treatment group compared to the placebo group between late January and February were observed consistently in both years, albeit statistically insignificance (OR = 0.69 in 2016 and OR = 0.75 in 2017); this effect is visibly striking during the peak URI months of January and February as graphically represented in the S6 Appendix. It is critical to remember that runny nose and cough are the primary mechanisms for spreading many respiratory infections.

As noted previously, the economic impact of the common cold is staggering with significant alternations in work attendance, productivity and caregiver absenteeism [3–6]. Given the results of our pilot study as well as the prevalence and importance of upper respiratory symptoms, further and more robust studies are necessary to evaluate the potential benefit of this low-cost and low-risk intervention.

## Supporting information

S1 Appendix Exclusion criteria and study procedure. (EML)

S2 Appendix Composition of the micronutrient and placebo caplets. (EML)

S3 Appendix Weekly questionnaire. (EML)

S4 Appendix Follow-up rates by treatement group. (EML)

S5 Appendix Primary analysis stratified by follow-up year. (EML)

S6 Appendix Statistical results for sensitivity analysis with adjustment for gender imbalance. (EML)

S1 Checklist CONSORT 2010 checklist of information to include when reporting a randomised trial*. (DOC)

S1 Protocol (DOCX)
