# Supplementary material for: The efficacy of a compounded micronutrient supplement on the incidence, duration, and severity of the common cold: A pilot randomized, double-blinded, placebo-controlled trial
Source: PLoS One. 2020 Aug 25;15(8):e0237491. doi: 10.1371/journal.pone.0237491 (PMC7447041; doi:10.1371/journal.pone.0237491)
Supplement: S1 Protocol — (DOCX) [file pone.0237491.s008.docx]

**RESEARCH PROTOCOL**

**THE EFFECT OF AN IMMUNE SYSTEM TARGETED MICRONUTRIENT SUPPLEMENT ON THE INCIDENCE, DURATION AND SEVERITY OF THE COMMON COLD: An expanded randomized, double-blinded, placebo-controlled trial**

**INTRODUCTION**

**Research question:**

**Does regular consumption of a multi-vitamin and mineral supplement composed of micronutrients essential to immune function reduce the incidence, duration and severity of the common cold?**

**THE COMMON COLD**

Although considered by many to be a nuisance illness, the common cold is the most prevalent illness worldwide. Most adults average 2 to 3 episodes per year; pre-school children average 7 episodes per year. Predominately caused by the pesky rhinovirus, it is estimated that there are 500 million to one billion episodes in the U.S. annually at direct costs of $17 billion and indirect costs of $2.5 billion. As many as 23 million lost workdays and 26 million lost school days occur each year due to colds. Indeed, the common cold is responsible for 40% of all time lost from work. Despite these significant statistics, the “cure” remains elusive.

**MICRONUTRIENTS AND IMMUNITY**

Research confirms that micronutrients play a vital role in immune system wellbeing, impacting both innate and adaptive immunity. Despite access to high quality foodstuffs in the United States, deficiencies in essential micronutrients are well documented. According to data from the National Health and Nutrition Examination Survey 2013 (NHANES 2013), significant percentages of the US population do not meet the estimated average daily nutritional requirements for the following micronutrients:

- 93% for Vitamin E
- 44% for Vitamin A
- 31% for Vitamin C
- 14% Vitamin B6
- 12% for Zinc.

Furthermore, Vitamin D deficiency is a major problem in the US and elsewhere; it has been estimated that 1 billion people worldwide have either vitamin D deficiency or insufficiency Holick, (2007). And according to the Linus Pauling Institute (LPI) at Oregon State University, micronutrient deficiencies are especially common amongst the elderly, impoverished, and over-nourished (obese), LPI (2015).

The Linus Pauling Institute also declares that “micronutrients play crucial roles in the development and expression of immune responses; selected micronutrient deficiencies can cause immunosuppression and thus increased susceptibility to infection and disease,” LPI (2015). Given the evidence for micronutrient role in immune health, it seems probable that individuals with a full complement of micronutrients essential for immunity might experience fewer, shorter and less intense episodes of the common cold.

**LITERATURE SEARCH**

LPI’s assertion notwithstanding, review of the healthcare literature dating from the 1970’s identifies but two RCTs comparing multi-vitamin and mineral supplementation versus placebo on the incidence, duration and severity of the common cold, Winkler (2005) and Barringer (2003).

Barringer found statistically significant reductions in the incidence and severity of the common cold amongst diabetics. Winkler’s trial failed to demonstrate statistically significant differences in incidence, duration or severity of the common cold, however.

Multiple studies pertaining to the benefits of vitamin C on the common cold have been published. Results are conflicting. Systematic reviews/meta-analyses conducted by Hemilä (2004), Van Straten (2002) and Douglas (2004) supported the beneficial effects for vitamin C, while the work of Sasazuki (2006), Audera (2001) and Hemilä (1997) failed to demonstrate benefit.

Several randomized-controlled trials and systematic reviews have documented the benefit of high dose zinc acetate lozenges in reducing the duration and severity of the common cold, notably: Petrus (1998), Prasad (2000), Science (2012), Hemilä (2011), Prasad (2008), and Hemilä (2015). High dose zinc gluconate has been found effective as well Mossad (1996) and zinc in combination with high dose vitamin C also ameliorates the symptoms of the common cold, according to Maggini (2012) and Wintergest (2005). However, long before much of the work cited above was published, Jackson (1997) concluded the evidence for the effectiveness of zinc salts lozenges in reducing the duration of the common cold was lacking.

In a blinded randomized controlled trial, Grimm (1999) found that Echinacea purpurea did not significantly decrease the incidence, duration and severity of the common cold.

**COMMUNITY HEALTH CARE RCT PILOT**

The Community Health Care Family Medicine Residency in collaboration with the University of Washington Center for Biostatistics conducted a double-blinded randomized clinical pilot comparing an immune system targeted micronutrient against placebo in the winter months of 2016. Sixty-one eligible participants were randomized into two groups (31 active v. 30 placebo). Study subjects took their assigned caplet daily for 90 days commencing January 4, 2016. Utilizing University of Washington Center for Biostatistics REDcap software, data was solicited weekly from participants for 120 days (January through April). No adverse events were reported.

Initial analysis of the data favors a substantial benefit for individuals randomized into the active group. This was especially pronounced in the month of February when the incidence of common cold symptoms was high. As of June 12, 2016 data analysis for the pilot is not finalized. The U of W Center for Biostatistics projects completion by the end of June 2016.

**RESEARCH PROPOSAL**

Based on the findings of the pilot study, we propose an expanded randomized, double-blinded, placebo-controlled trial, which investigates the incidence, severity and duration of the common cold comparing a carefully formulated immune system targeted multi-vitamin & mineral supplement against placebo.

Some features of the protocol include:

- Recruitment of up to 1000 eligible study subject participants from the South Puget Sound region aged 18 through 65 years
- Participant informed consent
- 180 day administration of active micronutrient v. placebo: October – March, 2017
- Participants agree to consume no other multivitamin and/or minerals during the study period
- 30 day multivitamin and mineral supplement abstinence beyond the administration of assigned caplet (April, 2017)
- Data solicited tracked for incidence, severity and duration October, 2016 through April, 2017
- Randomized, double-blinded, placebo-controlled
- Participants incentivized to complete data surveys weekly
- Community Health Care Family Medicine resident participation (Drs. LaPorte and Quackenbush)
- Approval through St. Joseph Hospital MREC IRB
- Approval by the Community Health Care Quality Improvement Committee
- Exclusion criteria: Pregnancy, breast-feeding, malabsorption syndromes, celiac disease, connective tissue disorders, immune deficiency, immune suppression, gastrointestinal surgery including bariatric surgery, cigarette smoking, kidney disease, liver disease, history for cancer and chemotherapy administration, and consumption of disease modifying anti-rheumatic drugs.
- Study director – James Lenhart, MD, MPH
- Research coordinator – TBD
  - Assists in recruitment of study subjects
  - Ensures subjects follow protocol
  - Tracks and monitors adverse events
- Conflicts of interest identified and mitigated (rigorous double-binding and external data analysis)
- University of Washington Center for Biostatistics facilitates study design and statistical analysis

**THE MICRONUTRIENT SUPPLEMENT FORMULA**

The active micronutrient formula, including dosages, is based on extensive information extracted from the Oregon State University Linus Pauling institute and the review of 23 peer-reviewed publications.

Ingredients were selected based on micronutrient’s evidence for critical role in immune support. The active ingredient supplement and placebo will be procured from DaVinci Laboratories, Burlington, VT <https://www.davincilabs.com> DaVinci Labs and its affiliates, strictly adhere to Supplement Good Manufacturing Practices.

**Here is the formula:**

Vitamin A: 2,500 IU as 50% retinol (retinyl palmitate or retinyl acetate) and 50% beta-carotene

Vitamin C: 1000 mg as L-ascorbic acid

Vitamin D: 2,000 IU as cholecalciferol (vitamin D3)

Vitamin E: 30 IU as RRR-alpha-tocopherol

Vitamin B6: 2 mg as pyridoxine hydrochloride

Vitamin B12: 30 µg as cyanocobalamin

Folate: 400 µg as folic acid

Zinc: 15 mg as zinc acetate

Selenium: 70 µg as sodium selenite

Copper: 900 µg as copper gluconate

To avoid the possible effects of interfering substances, the formula is:

- Gluten free
- Non-GMO
- Vegetarian
- No nuts
- No known interfering substances such as: citrate, glycine, tartrate, palm oil and cotton seed oil
- No artificial sweeteners
- Placebo mimics formula in taste, flavor, smell, feel and appearance.

**SUMMARY:**

The common cold is common and affects billions of people worldwide at costs estimated at over $20 billion annually in the United States. While zinc acetate lozenge formulations appear to reduce the duration and severity of this disorder, treatments to reduce frequency have been elusive.

This investigation seeks to expand on the pilot study previously referenced and answer the hypothesis: Does regular consumption of a multi-vitamin and mineral supplement composed of micronutrients essential to immune function reduce the incidence, duration and severity of the common cold?

**REFERENCES:**

Audera, C., 2001. Mega-dose vitamin C in treatment of the common cold: a randomized controlled trial. Med J Aust. 175(7): pp. 359-62.

Barringer, T., 2003. Effect of a multivitamin and mineral supplement on infection and quality of life: a randomized, double blinded, placebo-controlled trial. Annals of Internal Medicine. 138(5) pp.365-71.

Douglas, R., 2004. Vitamin C for preventing and treating the common cold. Cochrane Database Sys Rev. Oct 18;(4): CD000980.

Grimm, W., 1999. A randomized controlled trial of the effect of fluid extract of Echinacea purpurea on the incidence and severity of colds and respiratory infections.

Hemilä, H., 1997. Vitamin C intake and susceptibility to the common cold. Br J Nutr., 77(1): pp. 59-72.

Hemilä, H., 2004. Vitamin C supplementation and respiratory infections: a systematic review. Mil Med, 169(11): 920-5.

Hemilä, H., 2015. The effectiveness of high doses zinc acetate lozenges on various common cold symptoms: a meta-analysis. BMC Family Practice. 16(24) pp. 2-11.

Hemilä. H., 2011. Zinc may shorten the duration of colds: a systematic review. The Open Respiratory Medicine Journal. 5: pp.51-58.

Holick MF: Vitamin D deficiency. N Engl J Med 2007; 357(3): 266-81

<http://lpi.oregonstate.edu/mic> Accessed May 25, 2015.

Jackson, J., 1997. A meta-analysis of zinc salts lozenges and the common cold. Arch Intern Med. 157(20): pp. 2373-6.

Maggini, S., 2012. A combination of high-dose vitamin C plus zinc for the common cold. J Int Med Res. 40(1): pp. 28-42.

Mossad, S., 1996. Zinc gluconate lozenges for treating the common cold. A randomized, double-blind, placebo-controlled study. Ann Intern Med July 15; 125(2): pp. 81-88.

National Health and Nutrition Examination Survey, 2013. Online [available at]: <http://wwwn.cdc.gov/nchs/nhanes/search/nhanes13_14.aspx> [Accessed May 25, 2015].

Petrus, E., 1998. Randomized, double-masked, placebo-controlled clinical study of the effectiveness of zinc acetate lozenges on common cold symptoms in allergy-tested subjects. Current Therapeutic Research 59(9): pp. 594-607.

Prasad, A., 2000. Duration of symptoms and plasma cytokine levels in patients with the common cold treated with zinc acetate.

Prasad, A., 2008. Duration and severity of symptoms and levels of plasma interleukin-one receptor antagonists, soluble tumor necrosis factor receptor and adhesion molecules in patients with common cold treated with zinc acetate. JID, 197 (15 March) pp.795-802.

Sasazuki, S. et al, 2006. Effect of vitamin C on common cold: randomized controlled trial. Eur J Clin Nutr. 60(1): pp. 9-17.

Science, M., 2012. Zinc for the treatment of the common cold: a systematic review and meta-analysis of randomized controlled trials. CMAJ, 184 (10): E551-61.

The Linus Pauling Institute at Oregon State University, 2015. Online [available at]: <http://lpi.oregonstate.edu/mic> [Accessed May 25, 2015].

Van Straten, M., et al. 2002. Preventing the common cold with a vitamin C supplement: a double-blind placebo-controlled survey. Adv. Ther. May-June; 19(3): 151-159.

Winkler, P. 2005. Effect of a dietary supplement containing probiotic bacteria plus vitamins and minerals on common cold infections and cellular immune parameters. Int J Clin Pharmacol Ther43 (7): 318-26.

Wintergerst, E., 2006. Immune-enhancing role of vitamin C and zinc and effect on clinical conditions. Ann Nutr Metab. 50(2): pp. 85-94.

**APPENDIX: IMMUNE SYSTEM TARGETED MICRONUTRIENTS**

**Vitamin A**

**Agent:**

Vitamin A

**Role in immunity:**

*Innate and adaptive immunity:*

Helps maintain the structural and functional integrity of the mucosal cells of the eye, respiratory, gastrointestinal and genitourinary tracts.

Is important to the normal function of several types of immune cells including macrophages and neutrophils.

Vitamin A is needed for the proper function of cells that mediate adaptive immunity such as T and B cells and is necessary for the generation of antibody responses to specific antigens. Vitamin A plays a role in humoral and cell-mediated immunity. Vitamin A is also known as the anti-infective vitamin because it is required for normal functioning of the immune system.

**Nutrient interactions:**

Zinc deficiency is thought to interfere with vitamin A metabolism in several ways: decreased synthesis of retinol binding protein decreases the activity of the enzyme that releases the retinal from its storage form and is required for the enzyme that converts retinol to retinal.

**Agent and infectious disease:**

Vitamin A deficiency can be considered a nutritionally acquired immune deficiency disease. Children who are only mildly deficient in vitamin A have a higher incidence of respiratory disease and diarrhea as well as a higher rate of mortality from infectious diseases compared to children who consume sufficient vitamin A. Vitamin A supplementation has been found to decrease both the severity and incidence of death due to related diarrhea and measles in developing countries where vitamin A deficiency is common.

**Recommended dietary allowance:**

Adolescents aged 14 to 18 years 3000 IU for males 2333 IU for females

Adults aged 19 years and older 3000 IU for males 2333 IU for females

**NHANES survey:**

44% of the US population does not meet the estimated average daily requirement for vitamin A.

**Foods:**

Cod liver oil, fortified breakfast cereals, sweet potatoes, pumpkin, spinach, broccoli, butternut squash, carrots, and cantaloupe are rich sources.

**Supplements:**

Vitamin A supplementation enhances immunity and has been shown to reduce the infection related morbidity and mortality associated with its deficiency in diarrheal diseases, measles, malaria and HIV.

The principal forms of preformed vitamin A (retinol) in supplements are retinyl palmitate and retinyl acetate. Beta-carotene is also a common source of vitamin A in supplements.

**Safety/toxicity:**

Because of potential adverse effects, vitamin A supplementation should be reserved for the undernourished populations and those with evidence of vitamin A deficiency.

Vitamin A toxicity is called hypervitaminosis A. It is caused by overconsumption of preformed vitamin A, not carotenoids. Preformed vitamin A is rapidly absorbed and slowly cleared from the body.

Generally, signs of toxicity are associated with long-term consumption of vitamin A in excess of 25,000 to 33,000 IU per day. Some populations may be more susceptible to toxicity at lower doses, including the elderly, chronic alcohol users and people with genetic predisposition to high cholesterol.

**Tolerable upper intake level:**

Adolescence aged 14 to 18 years 9333 IU

Adults aged 19 years and older 10,000 IU

**LPI recommendation:**

Linus Pauline Institute recommends taking a multivitamin supplement that provides no more than 2,500 IU of preformed vitamin A (usually labeled as vitamin A acetate or vitamin A palmitate) and no more than 2,500 IU of additional vitamin A as beta-carotene (5,000 IU per day of vitamin A as retinol has been associated with adverse effects on bone health in older adults).

**Vitamin D**

**Agent:**

Vitamin D

**Role in immunity:**

Vitamin D3 modulates both *innate and adaptive immune responses*. Vitamin D3 functions as a steroid hormone to regulate expression of target genes. Many of the biological effects of 1,25-dihydroxy vitamin D3 are mediated through a nuclear transcription factor known as the vitamin D receptor or VDR. 1,25-dihydroxy vitamin D3 is now recognized to be a potent modulator of the immune system. The VDR is expressed in several types of immune cells, including monocytes, macrophages, dendritic cells and activated T cells.

Vitamin D has mainly inhibitory effects on adaptive immunity. In particular 1, 25-dihydroxy vitamin D3 suppresses antibody production by B cells and also inhibits proliferation of T cells.

Indeed, vitamin D deficiency has been implicated in the development of certain autoimmune diseases, such as insulin-dependent diabetes mellitus, multiple sclerosis and rheumatoid arthritis.

**Nutrient interactions:**

No nutrient interactions discussed.

The following medications may modify the metabolism of vitamin D: Dilantin, Tegretol, rifampin, cholestyramine and Orlistat.

**Agent and infectious disease:**

More than 200 viruses are responsible for causing infections of the upper respiratory tract known as the common cold resulting in symptoms of nasal congestion and discharge, cough, sore throat and sneezing. NHANES III reported an inverse relationship between serum 25 hydroxy vitamin D levels and recent self-reported URI symptoms. Compared to levels of circulating vitamin D equal to and above 30 ng/mL the risk of URI was 24% higher in individuals with levels between 10 and 29 ng per ml and 36% higher in those with levels below 10 ng per ml. A subgroup analysis indicated that low levels of serum 25-hydroxy vitamin D in subjects with asthma and chronic obstructive pulmonary diseases was linked to a greater susceptibility to URI when compared to people without pulmonary disease.

**Recommended dietary allowance:**

Adults aged 19 to 50 years 600 IU

Adults aged 51 to 70 years 600 IU

Adults aged 71 and older 800 IU

**NHANES survey:**

Vitamin D deficiency is a major problem in the US and elsewhere; it has been estimated that 1 billion people in the world have either vitamin D deficiency or insufficiency. Low environmental sunlight, concealed clothing, sun protection, skin pigmentation and exclusive breast-feeding are risk factors for vitamin D deficiency. Other risk factors include older age, chronic kidney disease, obesity, inflammatory bowel disease and magnesium deficiency.

Deficiency is assessed by measurement of total serum 25-hydroxy vitamin D. According to the US Endocrine Society, deficiency is less than 20 ng/ml, insufficiency is serum 25-dihydroxy vitamin D between 21 and 29 ng/ml and sufficiency as 25-hydroxy vitamin D values of 30 -100 ng/ml

**Foods:** primary source of vitamin D is sunlight. Salmon, sardines, milk, orange juice, fortified cereals and egg yolks are other sources.

**Supplements:**

Data from supplementation studies indicate that vitamin D intakes of at least 800 to 1000 IU per day are required by adults living in temperate latitudes to achieve serum 25-hydroxy vitamin D levels of at least 30 ng per ml.

Most vitamin D supplements are available without prescription and contain cholecalciferol (vitamin D3). Multivitamin supplements generally provide 400 to 1000 IU of vitamin D2 or vitamin D3. Supplementation with vitamin D2 or vitamin D3 is equally effective.

**Safety/toxicity:**

Vitamin D toxicity has not been observed to result from sun exposure. Over all, research suggests that vitamin D toxicity is very unlikely in healthy people at intake levels lower than 10,000 IU per day. Certain medical conditions can increase the risk of hypercalcemia in response to vitamin D including primary hyperparathyroidism, sarcoidosis, tuberculosis and lymphoma.

**Tolerable upper intake level:**

Adolescents aged 14 to 18 years 4000 IU

Adults aged 19 years and older 4000 IU

**LPI recommendation:**

The LPI recommends that generally healthy adults take 2000 IU of supplemental vitamin D daily.

**Vitamin C**

**Agent:**

Vitamin C

**Role in immunity:**

Vitamin C is a highly effective antioxidant that protects the body cells against reactive oxygen species that are generated by immune cells to kill pathogens. It is through this role that vitamin C affects several components of innate and adaptive immunity. Several studies have shown that supplemental vitamin C increases serum levels of antibodies. Some studies suggest the biological plausibility of vitamin C as an immune enhancer, human studies published to date are conflicting. Vitamin C has been shown to stimulate both the production and function of leukocytes.

**Nutrient interactions:**

No nutrient interactions were discussed, however, oral contraceptive pills and aspirin can lower vitamin C levels. It may also interact with Coumadin.

**Agent and infectious disease:**

A distinction has been observed between two groups of participants wherein regular supplementation with vitamin C did not reduce the incidence of colds in the general population, however, in participants undergoing heavy physical stress like marathon runners, vitamin C supplementation halved the incidence of colds. A benefit of regular vitamin C supplementation was also seen in the duration of colds with greater benefit in children than in adults. No significant effect of vitamin C supplementation was observed in therapeutic trials in which vitamin C was administered after cold symptoms occurred.

**Recommended dietary allowance:**

Adolescents aged 14 to 18 years 75 mg per day in males 65 mg per day in females

Adults 19 years and older 90 mg per day in males 75 mg per day in females

**NHANES survey:**

31% of the US population did not meet the estimated average requirement for vitamin C.

**Foods:**

Orange juice, grapefruit juice, oranges, grapefruits, strawberries, tomatoes, broccoli, and spinach are excellent sources.

**Supplements:**

L-ascorbic acid is available in many forms, but there is little scientific evidence than any one form is better absorbed or more effective than another.

**Safety/toxicity:**

There is no reliable scientific evidence that large amounts of vitamin C of up to 10 g per day in adults are toxic or detrimental to health

**Tolerable upper intake level:**

Adolescents aged 14 to 18 years 1800 mg per day

Adults 19 years and older 2000 mg per day

**LPI recommendation:**

Because of the very high benefit to risk ratio of vitamin C supplementation, and to ensure tissue and body saturation of vitamin C in almost all healthy people, the Linus Pauling Institute recommends of vitamin C intake of the least 400 mg daily for adult men and women. Consuming at least five servings of fruits and vegetables daily provides about 200 mg of vitamin C. Most multivitamin and mineral supplements provide 60 mg of vitamin C.

**Vitamin E**

**Agent:**

Vitamin E

**Role in immunity:**

Vitamin E is a lipid soluble antioxidant that protects the integrity of cell membranes from damaged caused by free radicals. Vitamin E supplementation in excess of current intake recommendations has been shown to enhance immunity and decrease susceptibility to certain infections especially in elderly individuals. More research is needed to determine whether supplemental vitamin E may protect the elderly against the common cold or other infections. Alpha-tocopherol is the form of vitamin E that appears to have the greatest nutritional significance. Severe vitamin E deficiency results mainly in neurological symptoms including impaired balance and coordination, injury to the sensory nerves and muscle weakness.

**Nutrient interactions:**

No nutrient interactions identified.

Drug interactions include warfarin, antiplatelet medications, Orlistat and cholestyramine.

**Agent and infectious disease:**

A randomized, placebo-controlled trial in elderly nursing home residents reported that daily supplementation with 200 international units of synthetic alpha-tocopherol for one year significantly lowered the risk of contracting upper respiratory tract infections, especially the common cold, but had no effect on lower respiratory tract infections.

**Recommended dietary allowance:**

Adolescents aged 14 to 18 years 15 mg or 22.5 IU in both males and females

Adults aged 19 years and older 15 mg or 22.5 IU in both males and females

**NHANES survey:**

27% of white participants, 41% of African-Americans, 28% of Mexican Americans, and 32% of the other participants were found to have blood levels of alpha-tocopherol less than 20 µmol per liter.

**Foods:**

Foods rich in vitamin E include: olive oil, soybean oil, corn oil, canola oil, sunflower oil, almonds, hazelnuts, spinach, carrots and avocados.

**Supplements:**

Supplements made from entirely natural sources contain only RRR-alpha-tocopherol. Many scientists believe it is difficult for an individual to consume more than 15 mg per day of alpha-tocopherol from food alone without increasing fat intake above recommended levels. The RRR-alpha-tocopherol is the isomeric preferred for use by the body, making it the most bioavailable form of alpha-tocopherol.

**Safety/toxicity:**

Few side effects have been noted in adults taking supplements of less than 2000 mg of alpha-tocopherol daily (RRR alpha-tocopherol). However, a meta-analysis that combined the results of 19 clinical trials of vitamin E supplementation for various diseases reported that adults who took supplements of 400 international units per day were 6% more likely to die from any cause than those who did not take vitamin E supplements. However, further analysis of the risk by vitamin E dose, and adjustment for other vitamin and mineral supplements, revealed that the increased risk of death was significant only at a dose of 2000 IU per day.

**Tolerable upper intake level:**

Adolescents aged 14 to 18 years 800 mg or 1200 IU

Adults aged 19 and older 1000 mg or 1500 IU

**LPI recommendation:**

LPI recommends generally healthy adults take a daily multivitamin mineral supplement. Most multivitamin supplements contain 30 IU of synthetic vitamin E or 90% of the RDA.

**THE B VITAMINS**

***VITAMIN B6***

**Agent:**

Vitamin B6

**Role in immunity:**

Deficiency in B6 has been shown to affect lymphocyte proliferation, differentiation and maturation as well as cytokine and antibody production. Correcting vitamin B6 deficiency restores the affected immune functions. Animal and human studies have demonstrated that vitamin B6 deficiency impairs aspects of adaptive immunity including both humoral and cell mediated immunity. There is evidence to suggest that adequate vitamin B6 intake is important for optimal immune system function, especially in older individuals. However, additional research is needed to evaluate whether vitamin B6 intakes higher than the current recommended dietary allowance could prevent and or reverse immune system impairments.

**Nutrient interactions:**

None listed

**Agent and infectious disease:**

See above

**Recommended dietary allowance:**

Adolescents aged 14 to 18 years 1.3 mg per day in males and 1.2 mg per day in females

Adults aged 19 to 50 years 1.3 mg per day for both males and females

Adults aged 51 years and older 1.7 mg per day four males and 1.5 mg per day for females

**NHANES survey:**

According to the data from the US National Health and Nutrition Examination Survey, 14% of the US population does not meet the estimated average requirement.

**Foods:**

Fortified cereals, turkey, avocado, chicken, bananas, dried plums, hazelnuts and spinach are good sources.

**Supplements:**

Vitamin B6 is available as pyridoxine hydrochloride in multivitamin, vitamin B complex and vitamin B6 supplements.

**Safety/toxicity:**

Long-term supplementation with very high doses of pyridoxine may result in painful neurological symptoms known as sensory neuropathy.

**Tolerable upper intake level:**

Adolescents aged 18 to 14 years 80 mg per day

Adults 19 years and older 100 mg per day

**LPI recommendation:**

LPI recommends that all adults take a daily multivitamin/mineral supplement, which usually contains at least 2 mg of vitamin B6. This amount is slightly above the RDA but still 50 times lower than the tolerable upper intake level set by the food and nutrition Board.

***FOLATE***

**Agent:**

Folic Acid

**Role in immunity:**

Folate deficiency results in impaired immune responses, primarily affecting cell mediated immunity. However, antibody responses of humoral immunity may also be impaired in folic acid deficiency.

**Nutrient interactions:**

Folate interacts with vitamin B12 and vitamin B6 to regulate the concentration of homocysteine in the blood. It is thought that vitamin C may limit degradation of natural folic acid and thus improve folic acid bioavailability. Also known to interact with aspirin, ibuprofen and some anticonvulsants like phenytoin.

**Agent and infectious disease:**

While folic acid is known to improve many neonatal outcomes like cleft palate, spina bifida and orofacial clefts as well as many cardiovascular disease effects, LPI made no specific mention of folate and infection or immune status.

**Recommended dietary allowance:**

Adolescents age 14 to 18 years 400 µg per day

Adults aged 19 years and older 400 µg per day

Pregnancy 600 µg per day (breast-feeding 500 µg per day)

**NHANES survey:**

No data provided

**Foods:**

Foods rich in folic acid include lentils, garbanzo beans, spinach, orange juice, white rice, enriched bread and orange juice.

**Supplements:**

The principal form of supplementary folate is folic acid.

**Safety/toxicity:**

No adverse effects have been associated with the consumption of excess folate from foods. Concerns regarding the safety are limited to synthetic folate intake.

**Tolerable upper intake level:**

Adolescents aged 14 to 18 years 800 µg per day

Adults 19 years and older 1000 µg per day

**LPI recommendation:**

The Linus Pauling Institute recommends that adults take a minimum daily multivitamin/mineral supplement, which typically contains 400 µg of folic acid

***B12***

**Agent:**

B12

**Role in immunity:**

Patients with diagnosed vitamin B12 deficiency have been reported to have suppressed natural killer cell activity and decreased numbers of circulating lymphocytes.

**Nutrient interactions:**

Enhances folate metabolism.

A number of drugs reduce the absorption of B12 including proton pump inhibitors, colchicine, metformin and cholestyramine.

**Agent and infectious disease:**

Apparently limited, see above.

**Recommended dietary allowance:**

Adolescents aged 14 – 18 years 2.4 mcg/day

Adults aged 19 and older years 2.4 mcg/day

**NHANES survey:** ND

**Foods:**

Clams, crab, beef, salmon, milk, turkey, and chicken are rich sources.

**Supplements:**

Cyanocobalamin is the principal form of vitamin B12 used in oral supplements.

**Safety/toxicity:**

No toxic or adverse effects have been associated with large intakes of vitamin B12 from food or supplements and healthy people.

**Tolerable upper intake level:**

Probably 2 mg per day.

**LPI recommendation:**

A varied diet should provide enough vitamin B12 to prevent deficiency in most individuals 50 years of age and younger. Strict vegetarians and women planning to become pregnant should take a multivitamin supplement daily, which would ensure an intake of 6 to 30 µg of B12.

**Zinc**

**Agent:**

Zinc

**Role in immunity:**

Zinc is critical for normal development and function of cells that mediate both the innate and adaptive immunity. The cellular function of zinc can be divided into three categories: catalytic, structural and regulatory. Zinc is not stored in the body. Therefore, regular dietary intake of the mineral is important in maintaining the integrity of the immune system. Thus, inadequate intake can lead to zinc deficiency and compromise immune responses. Even marginal zinc deficiency, which is more common than severe zinc deficiency, can suppress aspects of immunity. Zinc is a nutritionally essential mineral needed for catalytic, structural and regulatory functions in the body. Over 300 different enzymes depend on zinc. Zinc plays an important role in the structure of protein and cell membranes. Zinc also plays a role in cell signaling and has been found to influence hormone release and nerve impulse transmission and the ability to catalyze vital chemical reactions.

**Nutrient interactions:**

Taking large quantities of zinc (50 mg per day or more) over a period of weeks can interfere with copper bioavailability. Supplemental iron may decrease zinc absorption. High levels of dietary calcium impair zinc absorption in animals. The bioavailability of dietary folate is increased by the action of a zinc dependent enzyme. This suggests a positive interaction between zinc and folic acid.

Some drug interactions include tetracycline, quinolone antibiotics, bisphosphonates, and valproic acid.

**Agent and infectious disease:**

Adequate zinc intake is essential in maintaining the integrity of the immune system, specifically for normal development and function of cells that mediate both innate and adaptive immune responses. It also serves many antioxidant functions. Zinc deficient individuals are known to experience increased susceptibility to a variety of infectious diseases. These include diarrhea, pneumonia malaria and immune response in the elderly.

The Common Cold: the use of zinc lozenges within 24 hours of the onset of cold symptoms, and continued every 2 to 3 hours while awake until symptoms resolve, has been advocated for reducing the duration of the common cold. At least 10 controlled trials of zinc gluconate lozenges for the treatment of common colds and adults have been published. Five studies found that zinc lozenges reduced the duration of cold symptoms, whereas five studies found no difference between zinc lozenges and placebo lozenges with respect to the duration or severity of cold symptoms. A meta-analysis of published randomized controlled trials on the use of zinc gluconate lozenges found that evidence for their effectiveness in reducing the duration of common colds was still lacking.

A recent Cochrane review of 13 therapeutic trials found that, when taken within 24 hours of the onset of cold symptoms, zinc supplementation in the form of lozenges or syrup, reduced the severity and duration of cold symptoms. A systematic review and meta-analysis of 17 trials reported similar findings, but there was significant heterogeneity for the primary outcomes in both analyses. Moreover, another review found that beneficial effects on cold duration were seen in trials that provided more than 75 mg per day of zinc but not in trials that employed lower dosages. In trials that used high doses (greater than 75 mg per day) of zinc acetate, a 42% reduction in cold duration was observed.

**Recommended dietary allowance:**

The RDA for zinc in adults aged 19 years and older is 11 mg per day in males and 8 mg per day in females. Adolescents require approximately the same amount.

**NHANES survey:**

12% of the US population does not meet the estimated average requirement for zinc. Moreover, approximately 2,000,000,000 people worldwide are affected by dietary zinc deficiency, which leads to susceptibility to life-threatening infections in young children. Zinc deficiency has been estimated to cause more than 450,000 deaths in children under the age of five annually.

**Foods:**

Foods rich in zinc include oysters, beef, crab, turkey, beans, yogurt, cashews, almonds, peanuts and cheddar cheese.

**Supplements:**

Several forms are available and include zinc acetate, zinc gluconate, zinc picolinate and zinc sulfate. Zinc picolinate has been promoted as a more absorbable form of zinc, but there are few data to support this idea in humans

**Safety/toxicity:**

Short-term use of zinc lozenges (less than five days) has not resulted in serious side effects; bad taste and nausea were the most frequent adverse effects. In therapeutic trials, use of zinc lozenges for prolonged periods (6 to 8 weeks) is likely to result in copper deficiency.

Intranasal zinc is associated with anosmia and is not recommended.

**Tolerable upper intake level:**

In order to prevent copper deficiency, the US Food and Nutrition Board set the tolerable upper intake level for adults at 40 mg per day including dietary and supplemental zinc. Tolerable upper intake level is 34 mg per day in adolescents and 40 mg per day in adults

**LPI recommendation:**

The LPI recommends 15 mg per day daily of zinc as a multivitamin/mineral supplement.

**Selenium**

**Agent:**

Selenium

**Role in immunity:**

Adequate selenium intake is essential for the host to mount a proper immune response because it is required for the function of several selenium dependent enzymes known as selenoproteins**.** Selenium deficiency impairs aspects of innate as well as adaptive immunity, adversely affecting both humoral immunity (antibody production) and cell mediated immunity. Selenium deficiency appears to enhance the virulence or progression of some viral infections. Selenium supplementation in individuals who are not overtly deficient appears to stimulate the immune response.

**Nutrient interactions:**

No nutrient interactions listed. Valproic acid decreases plasma selenium levels.

**Agent and infectious disease:**

In two small studies, healthy and immunosuppressed individuals supplemented with 200 µg per day of selenium as sodium selenite for eight weeks showed an enhanced immune cell response to foreign antigens compared with those taking a placebo. Selenium deficiency appears to enhance the progression of some viral infections. The increased oxidative stress resulting from selenium deficiency may induce mutations or changes in the expression of some viral genes

**Recommended dietary allowance:**

Adolescents aged 14 to 18 years 55 µg per day

Adults aged 19 years and older 55 µg per day

**NHANES survey:**

Not on the NHANES list by LPI

**Foods:**

Shrimp, crab, halibut, rice, chicken, pork, beef, whole wheat bread, milk and walnuts are rich in selenium.

**Supplements:**

Sodium selenite and sodium selenate are inorganic forms of selenium. Selenate is almost completely absorbed, but a significant amount is excreted in the urine before it can be incorporated into proteins. Selenite is only about 50% absorbed, but it is better retained than selenate once it is absorbed. Selenomethionine an organic form of selenium that occurs naturally in foods is about 90% absorbed. Selenomethionine and selenium enriched yeast are also available as supplements.

**Safety/toxicity:**

Although selenium is required for health, like other nutrients, high doses of selenium can be toxic. Acute and fatal toxicities have occurred with accidental or suicidal ingestion of gram quantities of selenium. For example, clinically significant selenium toxicity was reported in 13 individuals after taking supplements that contained 27.3 mg per tablet due to a manufacturing error.

**Tolerable upper intake level:**

The Food and Nutrition Board of the Institute of Medicine recently set the tolerable upper intake for selenium at 400 µg per day in adults and adolescents. This includes selenium obtained from foods, which averages about 100 µg per day for adults in the US as well as some millennium from supplements.

**LPI recommendation:**

Eating a varied diet and taking a daily multivitamin supplement containing no more than 70 µg of selenium should provide sufficient selenium for most people in the US. Men taking supplemental selenium in order to reduce the risk of prostate cancer should not exceed 200 µg per day.

**Copper**

**Agent:**

Copper

**Role in immunity:**

Copper is known to play an important role in the development and maintenance of immune system function, but the exact mechanism of its action is not yet known. Neutropenia is a clinical sign of copper deficiency in humans. Adverse effects of insufficient copper on immune function appear most pronounced in infants. Moreover, 11 men on a low-copper diet (0.66 mg copper/day for 24 days and 0.38 mg/day for another 40 days) showed a decreased proliferation response when monocytes were isolated from their blood and presented with an immune challenge in cell culture. Recent mechanistic studies support a role for copper in innate immune response against bacterial infections. While severe copper deficiency has adverse effects on immune function, the effects of marginal copper insufficiency in humans are not yet clear.

**Nutrient interactions:**

Iron: Adequate copper nutritional status is necessary for normal iron metabolism and red blood cell formation.

Zinc: High supplemental zinc intakes of 50 mg/day or more for extended periods of time may result in copper deficiency. High dietary zinc intakes increase the synthesis of an intestinal cell protein called metallothionein, which binds certain metals and prevents their absorption by trapping them in intestinal cells. Metallothionein has a stronger affinity for copper than zinc, so high levels of metallothionein induced by excess zinc cause a decrease in copper absorption. In contrast, high copper intakes have not been found to affect zinc nutritional status.

**Agent and infectious disease:**

Not known. Zinc interferes with copper metabolism. Included to avoid the possible effects of this interaction.

**Recommended dietary allowance:**

Adolescents aged 14 to 18 years 890 µg per day

Adults aged 19 years and older 900 µg per day

**NHANES survey:**

Not on the NHANES list by LPI

**Foods:**

Liver, oysters, crab, cashews, sunflower seeds, almonds and lentils

**Supplements:**

Copper supplements are available as cupric oxide, copper gluconate, and copper sulfate.

**Safety/toxicity:**

Copper toxicity is rare in the general population. Acute copper poisoning has occurred through the contamination of beverages by storage in copper-containing containers, as well as from contaminated water supplies. Guideline values for copper in drinking water have been set by the US Environmental Protection Agency (1.3 mg/liter) and by the World Health Organization (2 mg/liter). Symptoms of acute copper toxicity include abdominal pain, nausea, vomiting, and diarrhea; such symptoms help prevent additional ingestion and absorption of copper

**Tolerable upper intake level:**

Adolescents aged 14 to 18 years 8,000 µg per day

Adults aged 19 years and older 10,000 µg per day

**LPI recommendation:**

The RDA for copper (900 mcg/day for adults) is sufficient to prevent deficiency, but the lack of clear indicators of copper nutritional status in humans makes it difficult to determine the level of copper intake most likely to promote optimum health or prevent chronic disease. A varied diet should provide enough copper for most people. For those who are concerned that their diet may not provide adequate copper, a multivitamin/mineral supplement will generally provide at least the RDA for copper.

**MICRONUTRIENT: IMMUNE SUPPORT DAILY SUPPLEMENT**

| **Micronutrient** | | **RDA (male/female)** | **Tolerable** | | **Linus Pauling Inst.** | **NHANES RDA** |
| --- | --- | --- | --- | --- | --- | --- |
| **Zinc** | | 11 mg/8 mg | 40 mg | | 15 mg | 12% USA |
| **Selenium** | | 55 µg | 400 µg | | 70 µg | ND |
| **Vitamin A** | | 3,000 IU/2,333 IU | 10,000 IU | | 2,500 IU/2,500IU | 44% USA |
| **Vitamin D** | | 600 U | 4,000 IU | | 2,000 IU | 1 billion WW* |
| **Vitamin C** | | 90 mg/75 mg | 2,000 mg | | 1000 mg | 31% USA |
| **Vitamin E** | | 15 mg or 22.5 IU | 1,500 IU | | 30 IU | 93% USA |
| **B6** | | 1.7 mg/1.5 mg | 100 mg | | 2 mg | 14% USA |
| **B12** | | 2.4 µg | 2 mg | | 6-30 µg | ND |
| **Folate** | | 400 µg | 1000 µg | | 400 µg | ND |
| **Copper** | 900 µg | | 10,000 µg | 900 µg | | ND |

* Estimated deficiency or insufficiency worldwide (not RDA)

THE FORMULA:

Vitamin A: 2,500 IU as 50% retinol (retinyl palmitate or retinyl acetate) and 50% beta-carotene

Vitamin C: 1000 mg as L-ascorbic acid

Vitamin D: 2,000 IU as cholecalciferol (vitamin D3)

Vitamin E: 30 IU as RRR-alpha-tocopherol

Vitamin B6: 2 mg as pyridoxine hydrochloride

Vitamin B12: 30 µg as cyanocobalamin

Folate: 400 µg as folic acid

Zinc: 15 mg as zinc acetate

Selenium: 70 µg as sodium selenite

Copper: 900 µg as copper gluconate
